# The Oxidation of Phytocannabinoids to Cannabinoquinoids

**DOI:** 10.1021/acs.jnatprod.9b01284

**Published:** 2020-04-21

**Authors:** Diego Caprioglio, Daiana Mattoteia, Federica Pollastro, Roberto Negri, Annalisa Lopatriello, Giuseppina Chianese, Alberto Minassi, Juan A. Collado, Eduardo Munoz, Orazio Taglialatela-Scafati, Giovanni Appendino

**Affiliations:** † Dipartimento di Scienze del Farmaco, Università del Piemonte Orientale, Largo Donegani 2, 28100 Novara, Italy; ‡ Maimonides Biomedical Research Institute of Córdoba; Department of Cellular Biology, Physiology and Immunology, University of Córdoba; University Hospital Reina Sofía, Avenida de Menendez Pidal s/n, 14004 Cordoba, Spain; § Dipartimento di Farmacia, Università di Napoli Federico II, Via Montesano 49, 80131 Napoli, Italy

## Abstract

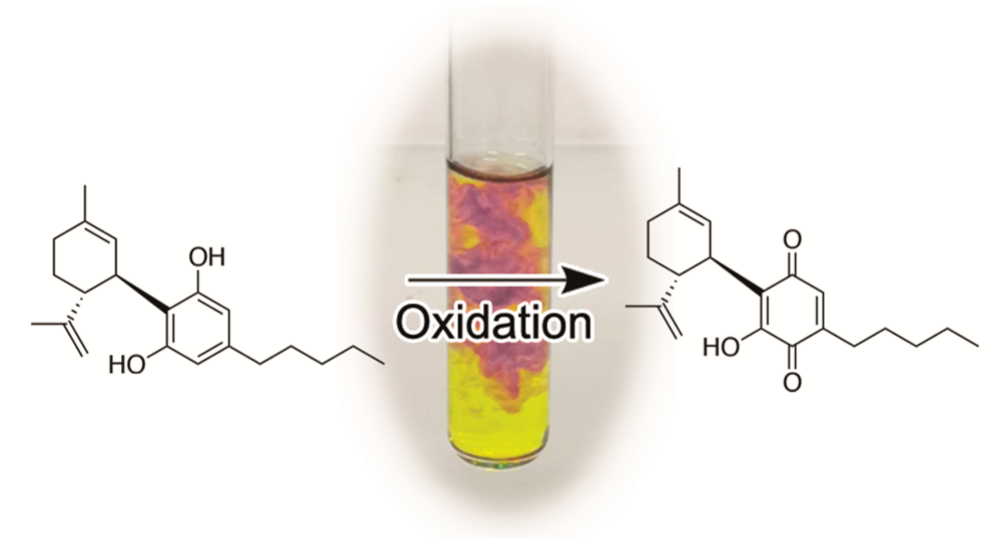

Spurred by a growing interest in cannabidiolquinone (CBDQ, HU-313, **2**) as a degradation marker and alledged hepatotoxic metabolite of cannabidiol (CBD, **1**), we performed a systematic study on the oxidation of CBD (**1**) to CBDQ (**2**) under a variety of experimental conditions (base-catalyzed aerobic oxidation, oxidation with metals, oxidation with hypervalent iodine reagents). The best results in terms of reproducibility and scalability were obtained with λ^5^-periodinanes (Dess-Martin periodinane, 1-hydroxy-1λ^5^,2-benziodoxole-1,3-dione (IBX), and SIBX, a stabilized, nonexplosive version of IBX). With these reagents, the oxidative dimerization that plagues the reaction under basic aerobic conditions was completely suppressed. A different reaction course was observed with the copper(II) chloride-hydroxylamine complex (Takehira reagent), which afforded a mixture of the hydroxyiminodienone **11** and the halogenated resorcinol **12**. The λ^5^-periodinane oxidation was general for phytocannabinoids, turning cannabigerol (CBG, **18**), cannabichromene (CBC, **10**), and cannabinol (CBN, **19**) into their corresponding hydroxyquinones (**20**, **21**, and **22**, respectively). All cannabinoquinoids modulated to a various extent peroxisome proliferator-activated receptor gamma (PPAR-γ) activity, outperforming their parent resorcinols in terms of potency, but the iminoquinone **11**, the quinone dimers **3** and **23**, and the haloresorcinol **12** were inactive, suggesting a specific role for the monomeric hydroxyquinone moiety in the interaction with PPAR-γ.

Color development has played a significat role in the early studies on Cannabis (*Cannabis sativa* L.) and cannabinoids. Thus, the first phytocannabinoids were purified from Cannabis red oil, a deep-red high-vacuum distillation fraction of Cannabis extracts.^1,2^ A red-purple color was also observed when fiber hemp or hashish was treated with methanolic KOH.^3^ Under these conditions, the development of a color is specific for Cannabis and Cannabis-derived products (marijuana, hashish),^4^ and the reaction has long been proved as an expeditious method for their identification in a forensic context (Beam test).^4^

The nature of the pigment from Cannabis red oil is still unclear, but color formation in the Beam test is the result of the aerobic oxidation of cannabidiol (CBD, **1**) to the hydroxyquinone **2** (cannabidiolquinone, CBDQ, HU-331),^5^ a compound that has attracted considerable interest because of its selective anticancer activity^6,7^ and catalytic inhibitory properties on topoisomerase IIα.^8^ While development of **2** as a drug was abandoned, possibly because of unfavorable stability properties (vide infra) and cellular toxicity,^9^ distinct lines of research rekindled interest in this compound. Thus, microsomial formation of **2** from CBD(**1**) has been associated with P450 covalent inhibition and perturbation of hepatic xenobiotics metabolism,^10^ and a similar process could also underlie the liver toxicity reported for high dosages of CBD.^11^ Furthermore, **2** is formed during long-term storage of CBD under aerobic conditions,^12^ and its availability is therefore important for quality control of this active pharmaceutical ingredient (API).
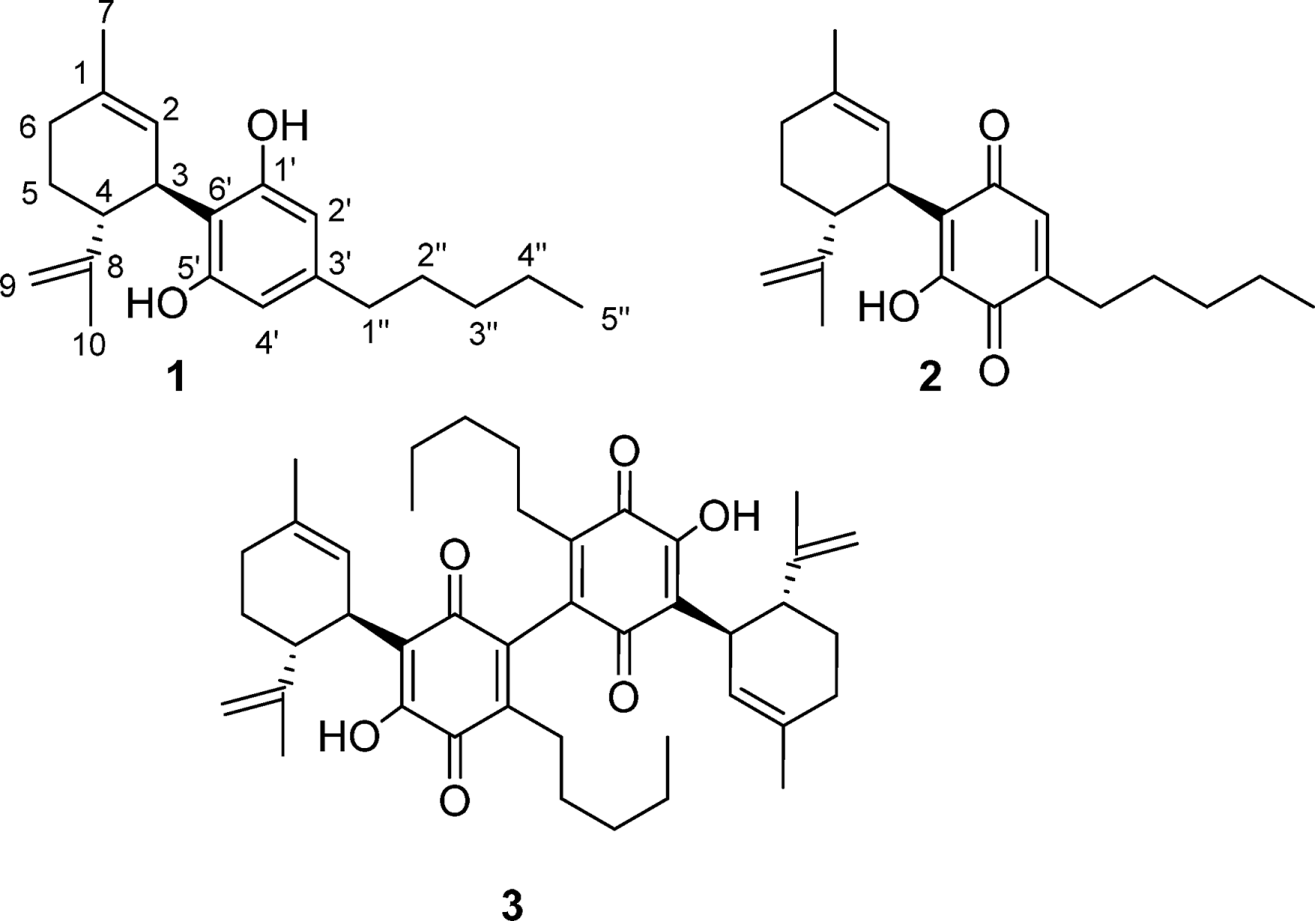


Despite the convergence of interest for CBDQ (**2**) from various areas of cannabinoid research, its only reported synthesis is the one inspired by the Beam test, that is, the aerobic oxidation of CBD in a cooled biphasic petroleum ether/5% ethanolic KOH system.^5,6^ Under these conditions, yields are erratic, scale-dependent, and modest (ca. 20% at best),^5,6^ while significant amounts of the dimeric quinone **3** are also formed by oxidative dimerization of CBDQ.^5^ Both reaction products, especially **3**, are unstable and rapidly turn into a complex mixture of polar compounds.^5^ In our hands, the oxidation reaction was poorly reproducible and could not be scaled up over a few hundred milligrams of starting material, even when air or 80% oxygen was bubbled into the biphasic reaction system. A more reproducible behavior was observed with KH or LiH in tetrahydrofuran (THF) or toluene under heterogeneous conditions, but scale-up was still problematic. While Beam-type oxidation strategies were eventually abandoned, their mechanism is worth mentioning. Thus, the reaction is presumably triggered by formation of a phenolate anion, next oxidized to an electrophilic radical (**4**) that adds to dioxygen to form a hydroperoxy radical. The latter is reduced to the corresponding anion (**5**) by a second phenolate ion, and, after tautomerization to **6**, the hydroperoxy anion is trapped by the *para*-carbonyl group. This generates the bridged keto-peroxyhemiacetal **7**, whose α-deprotonation triggers cleavage of the peroxidic bond, eventually affording the hydroxylated quinone **2** via the hydrate **8** (Figure 1).

**Figure 1:**
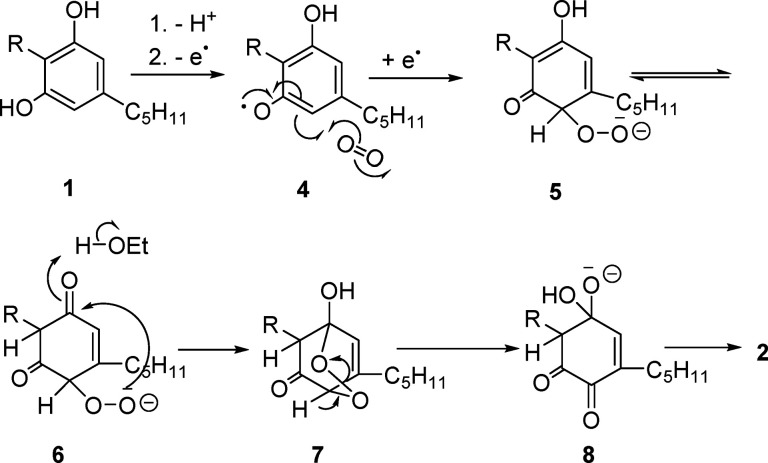
Possible mechanism of the base-mediated aerobic formation of cannabidiolquinone (CBDQ (**2**)) from cannabidiol (CBD (**1**)) in ethanolic KOH. (R = 3-*p*-mentha-1,8-dienyl).

This process is reminiscent of the transformation of vitamin K hydroquinone into its epoxyquinone form,^13^ and the mechanism outlined in Figure 1 could explain the sensitivity of the reaction to radical traps like butylated hydroxytoluene (BHT) as well as the unreactivity of monoalkylated phytocannabinoids, like Δ^9^-tetrahydrocannabinol (Δ^9^-THC, **9**) and cannabichromene (CBC, **10**), where the prototropic equilibrium required for the formation of the peroxyhemiacetal is not possible (cf. the formation of **6** from **5** in Figure 1).
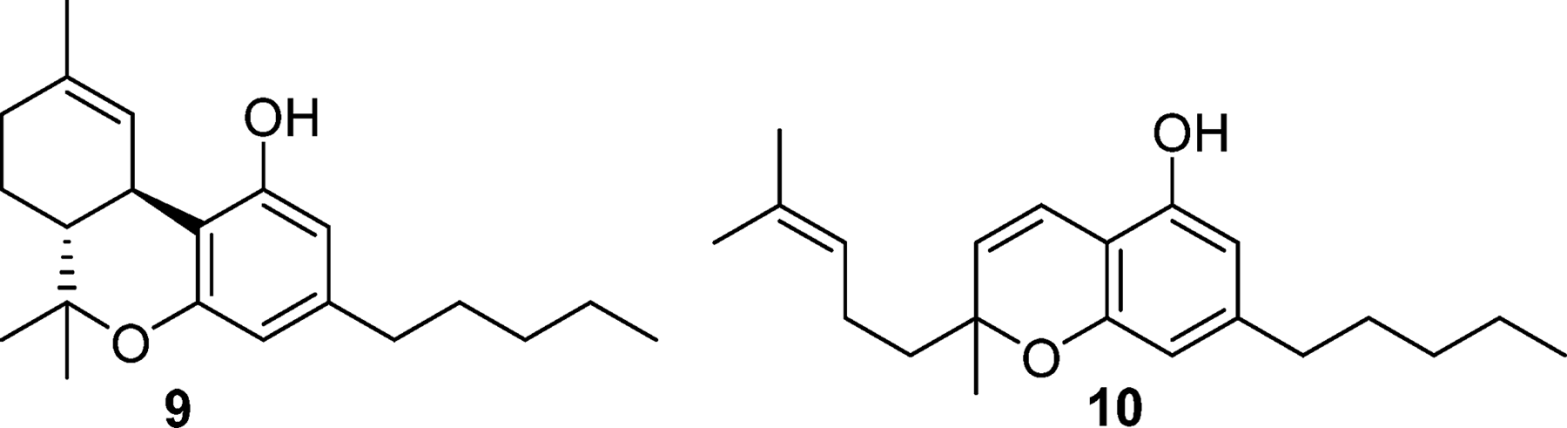


The reaction profile of the Beam test was basically replicated, without any substantial improvement of yield, by metal oxidants [FeCl_3_, K_3_[Fe(CN)_6_], MnO_2_, Cr^6+^-based reagents, CuCl, CuCl_2_, Ag_2_O, NH_4_Ce(NO_3_)_5_] under both catalytic and stoichiometric conditions, as well as by peroxides (*tert*-butyl hydroperoxide (TBHP), basic H_2_O_2_), with significant amounts of the dimer **3** being always formed under basic conditions or during the long reaction times required to achieve a significant conversion. A surprising and notable exception was the behavior of the Takehira complex (CuCl_2_-hydroxylamine),^14^ which afforded a mixture of the hydroxyiminodienone **11** and the chlororesorcinol **12**. The regioselectivity of the formation of **11** was deduced from the diagnostic ^3^*J* heteronuclear multiple bond correlation (HMBC) cross-peaks of H-1″ with the hydroxyiminocarbonyl carbon.
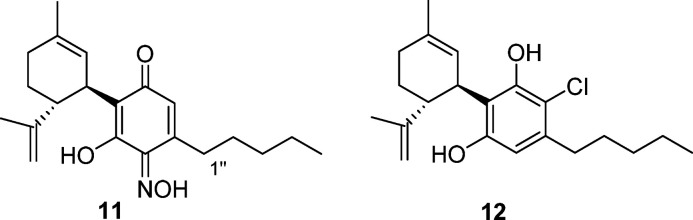


The Takehira complex was originally developed for the oxidation of methylpolyphenols to their corresponding hydroxyquinones,^14^ a reaction of relevance for the industrial synthesis of vitamin E,^14^ and was later modified by replacement of hydroxylamine with other nitrogen bases.^15^ In control experiments, copper(II) chloride alone gave CBDQ (**2**) and the dimer **3** as the only reaction products, while the quinone **2** did not react with hydroxylamine, suggesting a role for hydroxylamine in the chemoselective halogenation reaction, possibly via the generation of an *N*-chlorinated species, and of copper(II) in the activation of the quinonecarbonyl carbon toward nucleophilic attack by hydroxylamine.

Hypervalent iodine derivatives have become increasingly popular for a wide range of oxidative reactions,^16^ and bis(trifluoroacetoxy)iodobenzene (BTIB) was reported to oxidize the mono-*O*-alkylated cannabinoid Δ^9^-THC (**9**), otherwise unreactive in Beam-type oxidations,^6^ to its corresponding hydroxyquinone.^6^ This λ^3^-iodane was also able to oxidize CBD to CBDQ, but λ^5^ iodanes like 2-iodoxybenzoic acid (1-hydroxy-1λ^5^,2-benziodoxole-1,3-dione, IBX, **13**)^17^ and the Dess-Martin periodinane (DMP)^18^ gave much better and more reproducible yields, with a stabilized and not explosive version of IBX (SIBX)^19^ emerging as the reagent of choice. The superior behavior of SIBX compared to IBX might be related to the acidity of the stabilizing matrix (isophthalic and benzoic acids), which could help the hydrolytic cleavage of iodic esters formed in the reaction.^19^

The oxidation is presumably initiated by the sigmatropic rearrangement of the iodine–oxygen bond in the mixed λ^5^ iodane ester **14** formed by interaction of IBX and the C-1′ phenolic hydroxy group (Figure 2). The resulting C-2′ λ^3^-quinol **15**, after oxidation to the corresponding λ^5^-iodane **16**, is transformed by [3.3]-sigmatropic rearrangement of the carbon–oxygen bond into the C-4′ λ^5^-iodinane **17**, with β-elimination eventually generating the hydroxyquinone **2** and a reduced λ-iodinane. Remarkably, dimerization was completely suppressed under iodinane oxidation, and yields in the range of 50–60% could be obtained at multigram reaction scale. CBDQ, an orange powder,^20^ is unstable in solution, rapidly degrading in both protic (methanol) and aprotic (acetone, CHCl_3_) solvents, with generation of the more polar dimer **3** next to a host of uncharacterized more polar products. On the other hand, it could be stored for at least 10 months as a powder at −18 °C in a sealed flask, or for additional time as a frozen benzene or dimethyl sulfoxide (DMSO) solution at 4 °C.^21^

**Figure 2:**
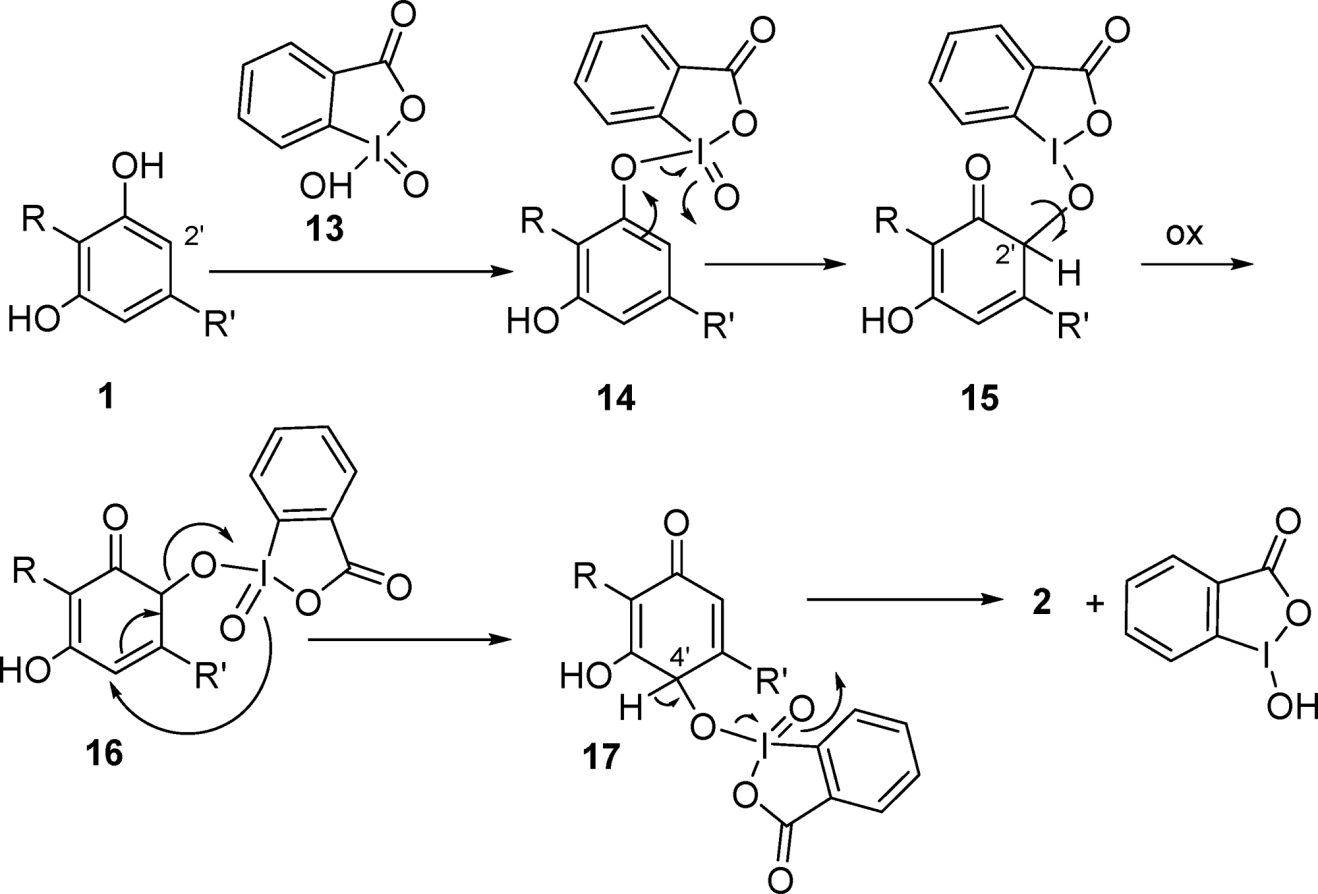
Possible mechanism for the SIBX-mediated formation of cannabidiolquinone (CBDQ (**2**)) from cannabidiol (CBD (**1**)) (R = 3-*p*-mentha-1,8-dienyl, R′ = *n*-pentyl).

The oxidation with SIBX is general for phytocannabinoids, and, apart from cannabigerol (**18**), it could also be applied to monoetherified compounds [cannabichromene (CBC, **10**), cannabinol (**19**)] that are unreactive under Beam-test conditions, to afford their corresponding hydroxyquinones **20**–**22**.
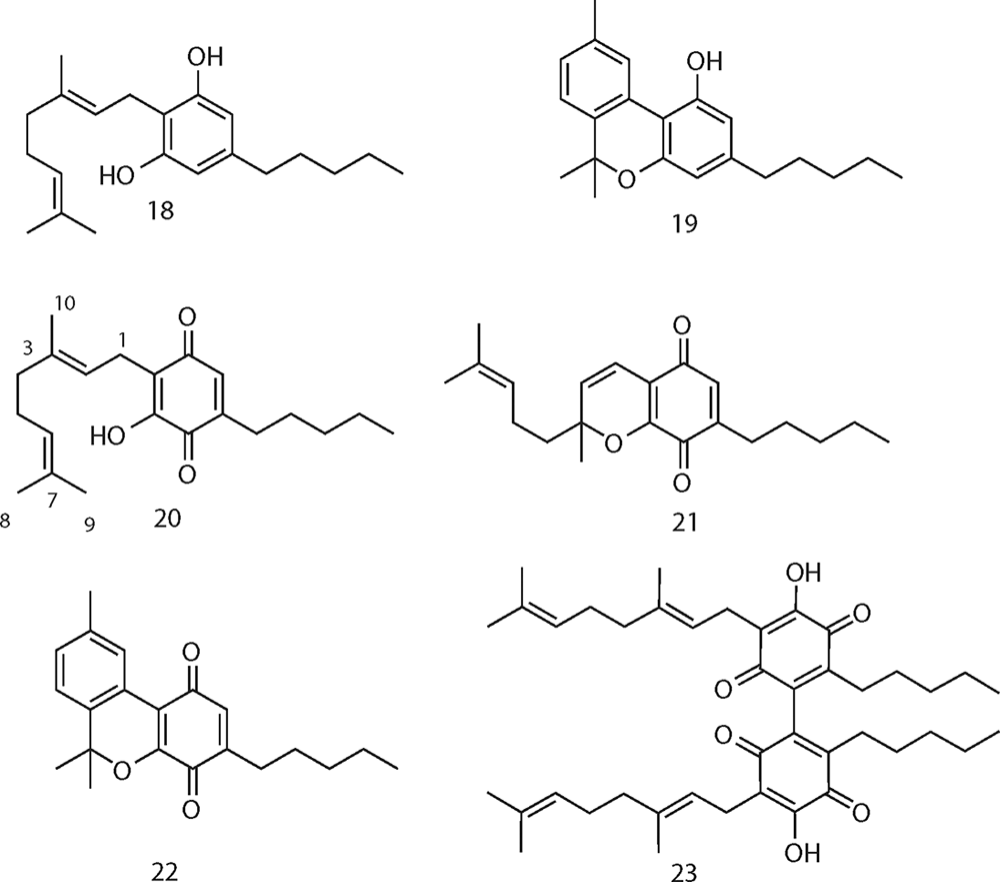


CBDQ (**2**) has been reported to be non-narcotic^6^ and lacks significant affinity for CB_1_ and CB_2_ receptors.^9^ Nevertheless, it showed powerful modulating activity on peroxisome proliferator-activated receptor gamma (PPAR-γ),^9,22^ and various degrees of PPAR-γ activating activity were also shown by the other cannabinoquinoids (Table 1). However, dimerization was detrimental for activity, and dimeric quinones were devoid of significant activity in PPARγ-activity assays.^23^ Dimeric quinones are axially chiral, and, since enantiomeric cannabinoids can show markedly different profiles of bioactivity,^2^ the one from CBG (CBGQ (**23**)) was resolved by chromatography on a chiral-phase column packed with amylose-tris(5-chloro-2-methylphenylcarbamate). However, both the (*aR*) and the (*aS*) enantiomers turned out to be inactive.^23^ Similarly, the hydroxyiminodienone **11** and the chlorinated resorcinol **12** were also devoid of activity.^23^

**Table 1:** PPAR-γ Modulation Activity^a^

compound	EC_50_
**1**	>25 μM
**10**	>25 μM
**18**	15.7 μM
**19**	>25 μM
**2**	10.5 μM
**21**	14.7 μM
**20**	4.9 μM
**22**	23.1 μM

a PPAR-γ modulation activity of the phytocannabinoids **1**, **10**, **18**, and **19** and their corresponding cannabinoquinones (**2**, **21**, **20**, and **22**). Rosiglitazone (1 μM) was used as positive control for PPAR-γ activation (50-fold induction over basal activity).

In conclusion, we have developed a reproducible and scalable synthesis of cannabinoquinoids, including CBDQ (**2**), significantly enhancing access to this compound^24^ of relevance not only for its bioactivity profile but also for the analytics of CBD, the study of its binding to P450 apoproteins, and its effects on liver function.

## Experimental Section

### General Experimental Procedures

IR spectra were recorded on an Avatar 370 FT-IR Techno-Nicolet apparatus. ^1^H (400 and 500 MHz) and ^13^C (100 and 125 MHz) NMR spectra were measured on Varian INOVA NMR spectrometers. Chemical shifts were referenced to the residual solvent signal (methanol-*d*_4_: δ_H_ = 3.34, δ_C_ = 49.0 or CDCl_3_: δ_H_ = 7.21, δ_C_ = 77.0). Homonuclear ^1^H connectivities were determined by the correlated spectroscopy (COSY) experiment. One-bond heteronuclear ^1^H–^13^C connectivities were determined with the heteronuclear single quantum coherence (HSQC) spectroscopy experiment. Two- and three-bond ^1^H–^13^C connectivities were determined by gradient two-dimensional (2D) heteronuclear multiple bond correlation (HMBC) experiments optimized for a ^2,3^*J* = 9 Hz. Low- and high-resolution electrospray ionization mass spectrometry (ESI-MS) data were determined on an LTQ OrbitrapXL (Thermo Scientific) mass spectrometer.

Reactions were monitored by thin-layer chromatography (TLC) on Merck 60 F254 (0.25 mm) plates, visualized by staining with 5% H_2_SO_4_ in EtOH and heating. Organic phases were dried with Na_2_SO_4_ before evaporation. Chemical reagents and solvents were purchased form Sigma-Aldrich and were used without further purification unless stated otherwise. Petroleum ether with boiling point of 40–60 °C was used. Silica gel 60 (70–230 mesh) was used for gravity column chromatography (GCC).

### SIBX Oxidation of Phytocannabinoids. Reaction with CBD (1) as Example

To a cooled (ice bath) solution of CBD (5 g, 15,6 mmol) in ethyl acetate (EtOAc, 75 mL), SIBX (21.1 g, 31.5 mmol, 2 molar equiv) was addd in six portions of ca. 5 g each. The cooling bath was removed, and the suspension was stirred at room temperature for 18 h and then filtered over a pad of diatomaceous earth. The filtration cake was washed with EtOAc (50 mL), and the pooled filtrates were washed with saturated Na_2_S_2_O_3_ (4 × 75 mL) and next with brine. After the drying and evaporation, the residue was purified by GCC on silica gel (75 g, petroleum ether–EtOAc 9:1 as eluant) to obtain a brown oil that solidified upon storing in the refrigerator. Washing with cold petroleum ether removed some of the colored impurities and afforded an orange powder (3.17 g, 61%). The same protocol was used for the oxidation and the purification of the other phytocannabinoids investigated (CBC, **10**; CBG, **18**; CBN, **19**). The scale was 100–200 mg, and the yields were 59, (CBCQ, **21**), 37 (CBGQ, **20**), and 58%, (CBNQ, **22**).

#### Cannabigeroquinone (CBGQ, **20**)

Red powder, IR ν_max_ (KBr disc): 3272, 2955, 2923, 2856, 1644, 1637, 1350, 1316, 1191, 1175, 580 cm^–1^; ^1^H NMR (CDCl_3_, 400 MHz) δ 6.94 (1H, s, OH), 6.45 (1H, bs, H-2′), 5.13 (1H, t, *J* = 7.4 Hz, H-2), 5.04 (1H, t, *J* = 6.7 Hz, H-7), 3.13 (2H, d, *J* = 7.4 Hz, H-1), 2.41 (2H, t, *J* = 7.6, H-1″), 1.99–1.90 (4H, m, H-4, H-5), 1.73 (3H, s, H-8), 1.64 (3H, s, H-9), 1.57 (3H, s, H-10), 1.50 (2H, m, H-2′′), 1.33 (4H, m, H-3′′, H-4′′), 0.89 (3H, t, *J* = 6.8 Hz, H-5″); ^13^C NMR (CDCl_3_, 100 MHz) δ 187.7, 184.2, 150.9, 145.1, 137.3, 134.4, 131.5, 124.3, 120.2, 119.7, 39.8, 31.5, 28.3, 27.4, 26.7, 25.8, 22.5, 22.0, 17.8, 16.3, 14.0; ESI-MS: *m*/*z* 331 [M + H]^+^; high-resolution (HR) ESI-MS *m*/*z* 331.2262 [M + H]^+^, calcd. for C_21_H_31_O_3_, 331.2268.

#### Cannabichromenquinone (CBCQ, **21**)

Red oil, IR ν_max_ (KBr disc): 2957, 2926, 2852, 1648, 1580, 1324, 1078, 969, 891 cm^–1^; ^1^H NMR (CDCl_3_, 400 MHz) 6.47 (1H, d, *J* = 9.9 Hz, H-1), 6.40 (1H, bs, H-2′), 5.56 (1H, d, *J* = 9.9 Hz, H-2), 5.07 (1H, t, *J* = 6.9 Hz, H-6), 2.39 (2H, t, *J* = 7.6 Hz, H-1″), 2.08 (1H, m, H-5a), 1.88 (1H, m, H-5b), 1.66 (2H, overlapped, H-4), 1.64 (3H, s, H-8), 1.55 (3H, s, H-9), 1.49 (2H, m, H-2′′), 1.46 (3H, s, H-10), 1.32 (4H, m, H-3′′, H-4′′), 0.89 (3H, t, *J* = 6.7 Hz, H-5″); ^13^C NMR (CDCl_3_, 100 MHz) δ 184.6, 181.9, 150.8, 147.7, 132.2, 131.4, 128.8, 123.4, 115.4, 115.0, 83.0, 41.5, 31.4, 28.7, 27.4, 27.3, 25.6, 22.6, 22.4, 17.7, 13.9; ESI-MS *m*/*z* 329 [M + H]^+^; HR ESI-MS *m*/*z* 329.2107 [M + H]^+^, calcd for C_21_H_29_O_3_, 329.2111.

#### Cannabinolquinone (CBNQ, **22**)

Red oil, IR ν_max_ (KBr disc): 2955, 2924, 2855, 1649, 1382, 1145, 1110, 811 cm^–1^; ^1^H NMR (CDCl_3_, 400 MHz) δ 8.30 (1H, s, H-2), 7.09 (1H, d, *J* = 7.9 Hz, H-6), 7.02 (1H, d, *J* = 7.9 Hz, H-5), 6.63 (1H, t, *J* = 1.4 Hz, H-2′), 2.40 (2H, t, *J* = 7.7 Hz, H-1″), 2.36 (3H, s, H-7), 1.69 (6H, s, H-9, H-10), 1.56 (2H, m, H-2″), 1.32 (4H, m, H-3′′, H-4′′), 0.90 (3H, t, *J* = 6.8 Hz, H-5″); ^13^C NMR (CDCl_3_, 100 MHz) δ 180.2, 175.3, 163.3, 144.7, 138.1, 133.8, 131.8, 128.9, 125.7, 122.3, 111.0, 82.7, 53.6, 31.6, 29.8, 29.0, 28.3, 27.4, 22.4, 21.4, 13.9; ESI-MS *m*/*z* 325 [M + H]^+^; HR ESI-MS *m*/*z* 325.1791 [M + H]^+^, calcd. for C_21_H_25_O_3_, 325.1798.

### Dimeric Cannabigeroquinone (23) and Chiral-Phase Chromatography

Red powder, IR ν_max_ (KBr disc): 3280, 2955, 1350, 1188, cm^–1^; ^1^H NMR (CDCl_3_, 400 MHz) δ 6.98 (1H, s, OH), 5.17 (1H, t, *J* = 7.4 Hz, H-2), 5.06 (1H, t, *J* = 6.7 Hz, H-7), 3.16 (2H, d, *J* = 7.4 Hz, H-1), 2.32 (2H, t, *J* = 7.6, H-1″), 2.05–1.90 (4H, m, H-4, H-5), 1.73 (3H, s, H-8), 1.64 (3H, s, H-9), 1.57 (3H, s, H-10), 1.49 (2H, m, H-2′′), 1.32 (4H, m, H-3′′, H-4′′), 0.89 (3H, t, *J* = 6.8 Hz, H-5″). ESI-MS: *m*/*z* 645 [M + H]^+^; HR ESI-MS *m*/*z* 645.4159 [M + H]^+^, calcd. for C_41_H_57_O_6_, 645.4155.

A sample of compound **23** (2.0 mg) was separated on a chiral-phase Lux 5 μ Amylose-2 250 × 4.60 mm column, Phenomenex, eluent *n*-hexane/isopropyl alcohol 9:1 (0.2% trifluoroacetic acid (TFA)) with a flow of 0.7 mL/min, and two peaks were obtained with *R*_t_ = 8 min (0.9 mg) and *R*_t_ = 13 min (0.7 mg).

### Oxidation of Cannabidiol (CBD, 1) with the Takehira Reagent

To a stirred solution of CBD (**1**, 200 mg, 0,64 mmol) in toluene–*tert*-butanol (3:1, 20 mL), copper(II) chloride (43 mg, 0.32 mmol, 0.5 molar equiv) and hydroxylamine hydrochloride (22 mg, 0.32 mmol, 0,.5 molar equiv) were added. The solution turned from yellow to brown and was stirred for 2 h at rt, worked up by dilution with 2N H_2_SO_4_ and extraction with EtOAc. The organic phase was washed with brine, dried with Na_2_SO_4_, filtered, and evaporated. The residue was purified by GCC (5 g silica gel, petroleum ether–EtOAc gradient, from to petroleum ether to 95:5 petroleum ether–EtOAc as eluent) to give **12** (135 mg, 34%) and **11** (20%).

#### Hydroxyiminocannabiquinone (**11**)

Brownish oil, IR ν_max_ (KBr disc): 2960, 2924, 2856, 1617, 1420, 1420, 1260, 1092, 1016, 797 cm^–1^; ^1^H NMR (methanol-*d*_4_, 400 MHz) δ 6.25 (1H, s, H-2′), 5.12 (1H, s, H-2), 4.50 (1H, s, H-9a), 4.49 (1H, s, H-9b), 3.82 (1H, m, H-3), 2.92 (1H, td, *J* = 11.7, 3.2 Hz, H-4), 2.70 (2H, t, *J* = 7.5 Hz, H-1″), 2.18 (1H, m, H-6a), 1.99 (1H, m, H-6b), 1.73 (2H, overlapped, H-5), 1.65 (3H, s, H-7), 1.63 (3H, s, H-10), 1.60 (2H, overlapped, H-2′′), 1.34 (2H, overlapped, H-3′′), 1.33 (2H, overlapped, H-4′′), 0.91 (3H, t, *J* = 6.9 Hz, H-5″); ^13^C NMR (methanol-*d*_4_, 100 MHz) δ 176.3 (C-1′), 168.1 (C-5′), 150.2 (C-8), 148.7 (C-3′), 148.1 (C-4′), 133.6 (C-1), 125.6 (C-2), 120.6 (C-2′), 119.1 (C-6′), 110.8 (C-9), 45.6 (C-4), 36.2 (C-3), 32.8 (C-3″), 31.6 (C-6), 31.5 (C-1′′), 30.8 (C-5), 30.5 (C-2′′), 23.6 (C-7), 23.5 (C-4′′), 19.1 (C-10), 14.3 (C-5′′); ESI-MS *m*/*z* 344 [M + H]^+^; HR ESI-MS *m*/*z* 344.2210 [M + H]^+^ calcd for C_21_H_30_NO_3_, 344.2220.

#### 2-Chlorocannabidiol (12)

Yellow oil, IR ν_max_ (KBr disc): 3500, 3421, 2962, 2924, 2859, 1623, 1421, 1258, 1193, 1054, 888, 817, 698 cm^–1^; ^1^H NMR (methanol-*d*_4_, 400 MHz): δ 6.21 (1H, s, H-2′), 5.25 (1H, s, H-2), 4.46 (1H, s, H-9a), 4.44 (1H, s, H-9b), 3.99 (1H, m, H-3), 2.95 (1H, m, H-4), 2.56 (2H, t, *J* = 7.4 Hz, H-1″), 2.20 (1H, m, H-5a), 2.01 (1H, d, *J* = 17.1 Hz, H-5b), 1.76 (2H, m, H-6), 1.68 (3H, s, H-7), 1.65 (3H, s, H-10), 1.56 (2H, m, H-2″), 1.35 (4H, m, H-3′′-4′′), 0.91 (3H, t, *J* = 6.8 Hz, H-5″); ^13^C NMR (methanol-*d*_4_, 100 MHz): δ 156.1, 152.7, 150.2, 139.3, 134.2, 126.6, 118.2, 112.8, 110.7, 109.6, 46.2, 38.3, 34.7, 32.7, 31.7, 30.7, 30.5, 23.7, 23.5, 19.3, 14.4. ESI-MS *m*/*z* 349, 351 [M + H]^+^ ratio 3:1; HR ESI-MS *m*/*z* [M + H]^+^349.1919 (calcd for C_21_H_30_^35^ClO_2_, 349.1929).

### PPAR-γ Activity Evaluation

Human embryonic kidney epithelial cells 293T cells were obtained from the American Type Culture Collection (CRL-3216) and cultured in Dulbecco’s Modified Eagle’s Medium (DMEM) supplemented with 10% fetal calf serum (FCS) and antibiotics. To analyze the PPAR-γ transcriptional activity, HEK-293T cells were cultured in 24-well plates (2 × 104 cells/well) and transiently cotransfected with GAL4-PPAR-γ (50 ng) and GAL4-luc (firefly luciferase, 50 ng) vectors using Roti-Fect (Carl Roth). Twenty hours after transfection the cells were stimulated with increasing concentrations of the compounds for 6 h, and luciferase activities were quantified using Dual-Luciferase Assay (Promega). Rosiglitazone (1 μM, Cayman Chemical), was used as a positive control for PPAR-γ activation (50-fold induction over basal activity). Test compounds and controls stocks were prepared in DMSO, and the final concentration of the solvent was always less than 0.5% v/v. The plasmid GAL4-PPAR-γ was obtained from Prof. C. Sinal (Dalhousie University). Half-maximal effective concentration (EC_50_) was estimated using Prism software (GraphPad). All transfection experiments were performed at least three times.

## Supporting Information Available

^1^H and ^13^C NMR spectra for CBDQ (**2**) and the other cannabinoquinoids mentioned in this study (PDF)
