# Supplementary material for: The Oxidation of Phytocannabinoids to Cannabinoquinoids
Source: J Nat Prod. 2020 Apr 21;83(5):1711–5. doi: 10.1021/acs.jnatprod.9b01284 (PMC7997633; doi:10.1021/acs.jnatprod.9b01284)
Supplement: Supplementary file 1 — np9b01284_si_001.pdf [file np9b01284_si_001.pdf]

SUPPORTING INFORMATION

The Oxidation of Phytocannabinoids to Cannabinoquinoids

Diego Caprioglio,[†] Daiana Mattoteia,[†] Federica Pollastro,[†] Annalisa Lopatriello, [§] Giuseppina Chianese, [§] Alberto Minassi,[†] Eduardo Munoz,[‡] Orazio Taglialatela-Scafati,[*§] and Giovanni Appendino [*†]

[†] Dipartimento di Scienze del Farmaco, Università del Piemonte Orientale, Largo Donegani 2, 28100, Novara, Italy

[‡] Maimonides Biomedical Research Institute of Córdoba; Department of Cellular Biology, Physiology and Immunology, University of Córdoba; University Hospital Reina Sofía. Avenida de Menendez Pidal s/n, 14004 Cordoba, Spain

[§] Dipartimento di Farmacia, Università di Napoli Federico II, Via Montesano 49, 80131 Napoli, Italy

Contents:

Figure S1. ^{1}H NMR spectrum (400 MHz) of compound **11** in CD_3OD ……………………Page 3

Figure S2. ^{13}C NMR spectrum (100 MHz) of compound **11** in CD_3OD…………………… Page 3

Figure S3. ^{1}H-^{1}H COSY spectrum of compound **11** …………………………………………….Page 4

Figure S4. 2D NMR HSQC spectrum of compound **11**………………………………………… Page 4

Figure S5. 2D NMR HMBC spectrum of compound **11**…………………………………………Page 5

Figure S6. ^{1}H NMR spectrum (400 MHz) of compound **12** in CD_3OD……………………Page 6

Figure S7. ^{13}C NMR spectrum of compound **12** in CD_3OD……………………………………Page 6

Figure S8. ^{1}H NMR spectrum (500 MHz) of compound **20** in $CDCl_3$ ………………………………. Page 7

Figure S9. ^{13}C NMR spectrum of compound **20** in $CDCl_3$ …………………………………………………Page 7

Figure S10. ^{1}H NMR spectrum (400 MHz) of compound **21** in $CDCl_3$……………………………….Page 8

Figure S11. ^{13}C NMR spectrum of compound **21** in $CDCl_3$ ……………………………………………… Page 8

Figure S12. ^{1}H NMR spectrum (400 MHz) of compound **22** in $CDCl_3$………………………………. Page 9

Figure S13. ^{13}C NMR spectrum of compound **22** ……………………………………………………………Page 9

Figure S14. ^{1}H NMR spectrum (400 MHz) of compound **23** in $CDCl_3$ ……………………………..Page 10

Figure S15. CD spectra of the chromatographic peaks of compound **23**…………………………Page 10

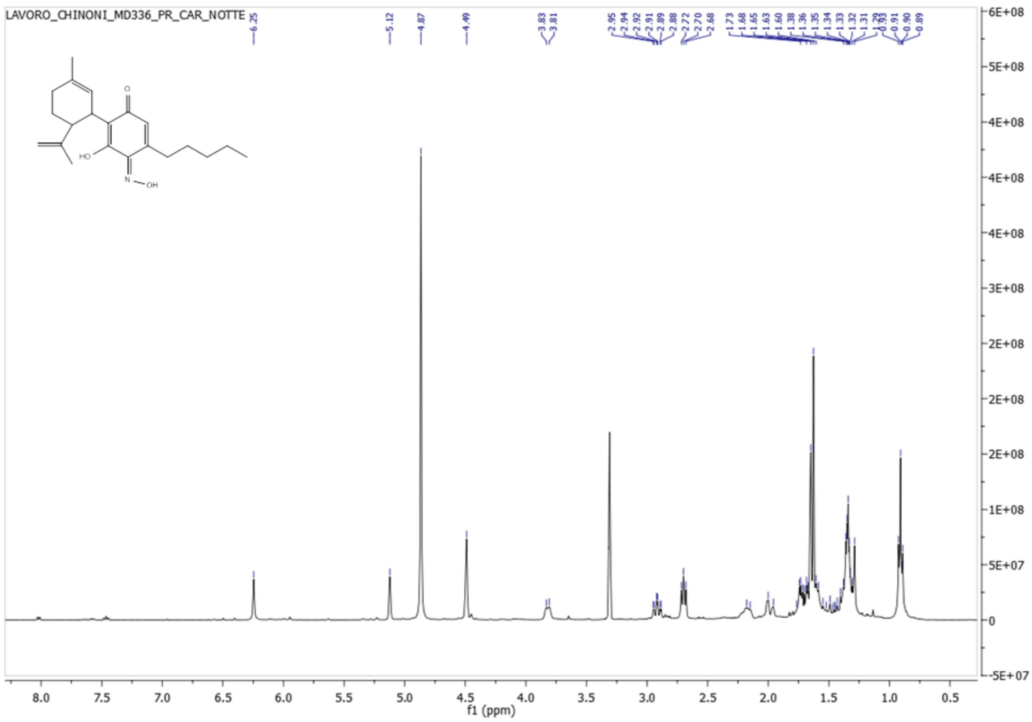

Figure S1. ^{1}H NMR spectrum (400 MHz) of compound **11** in CD_3OD

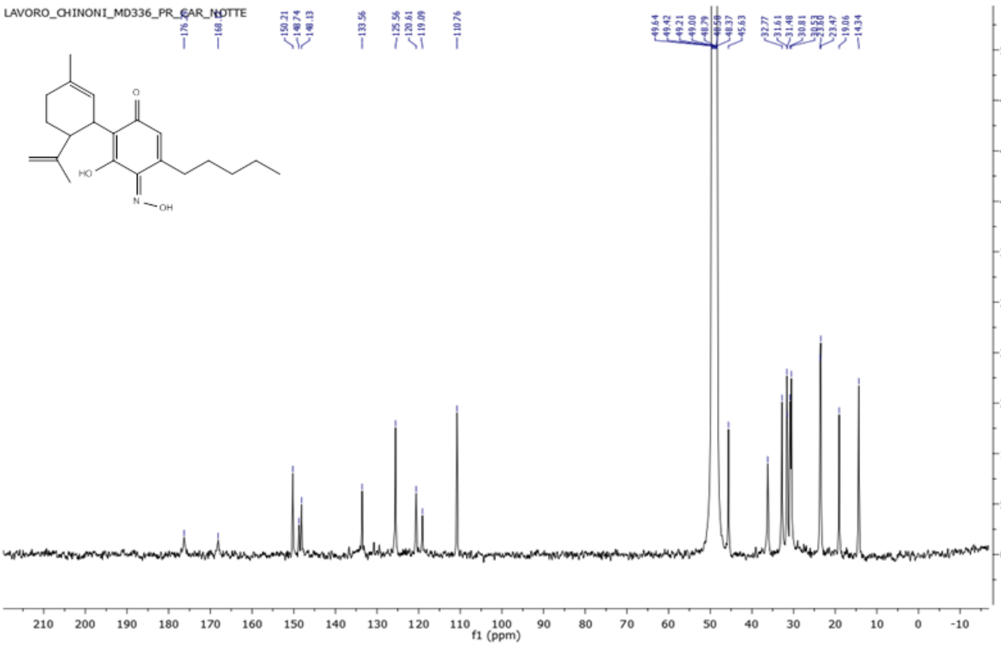

Figure S2. ^{13}C NMR spectrum (100 MHz) of compound **11** in CD_3OD

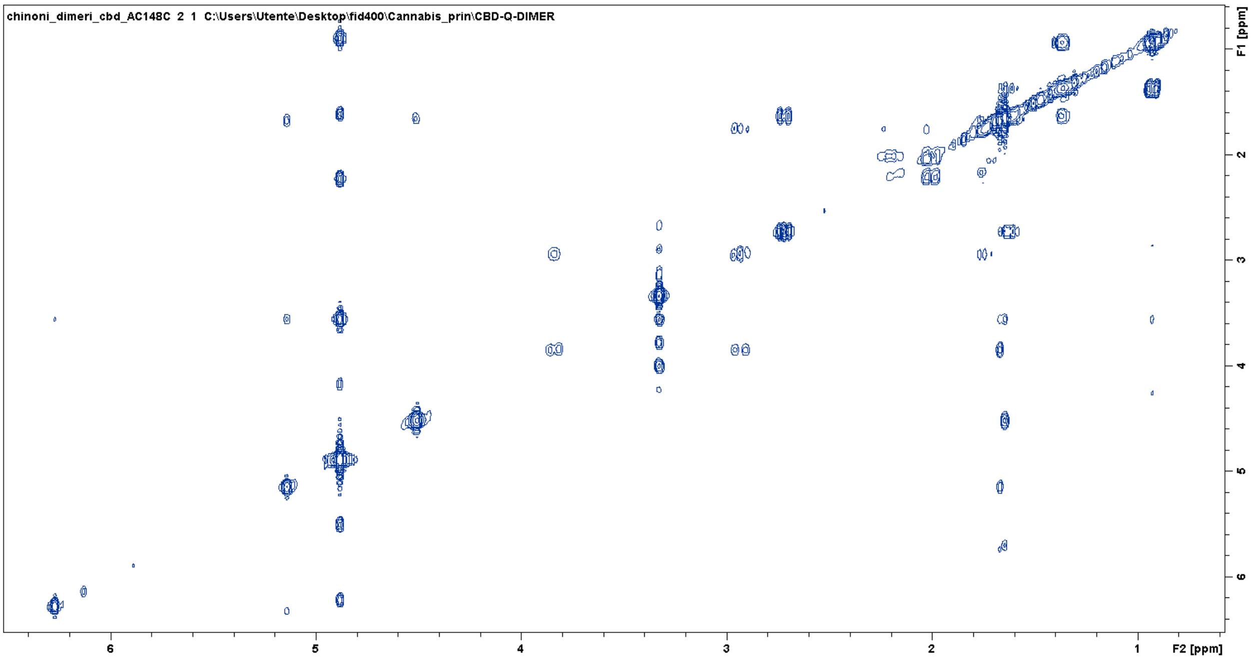

Figure S3. ^{1}H-^{1}H COSY spectrum of compound **11**

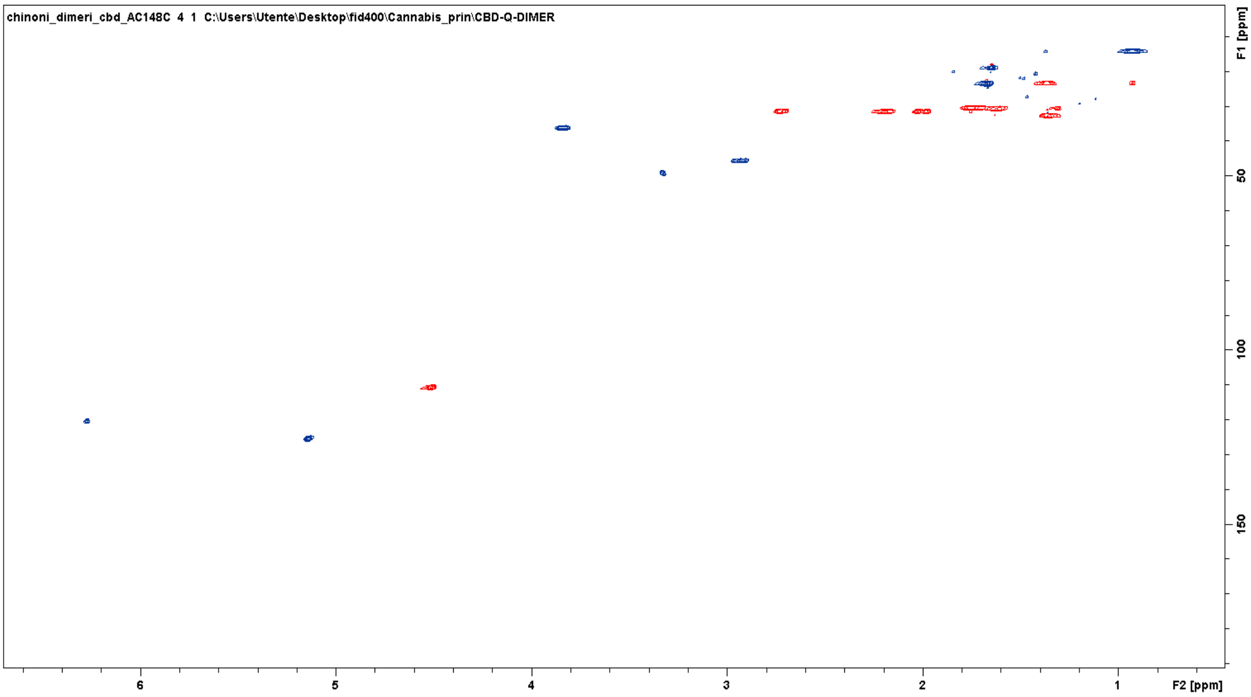

Figure S4. 2D NMR HSQC spectrum of compound **11**

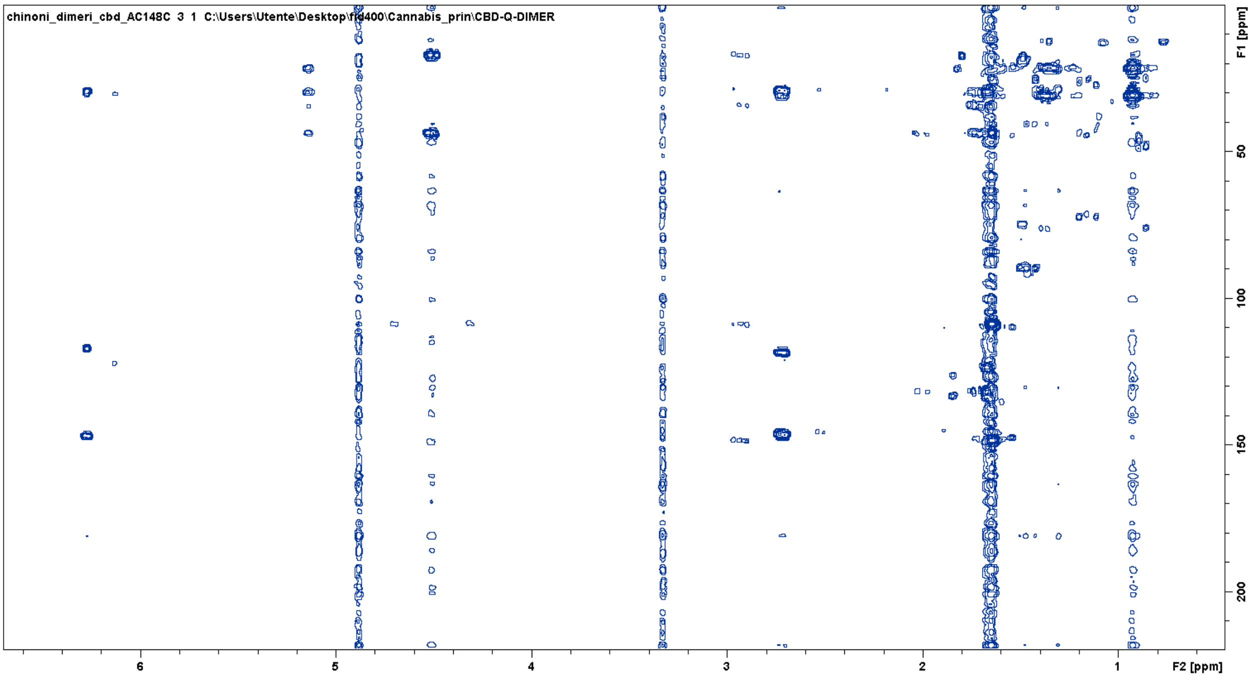

Figure S5. 2D NMR HMBC spectrum of compound **11**

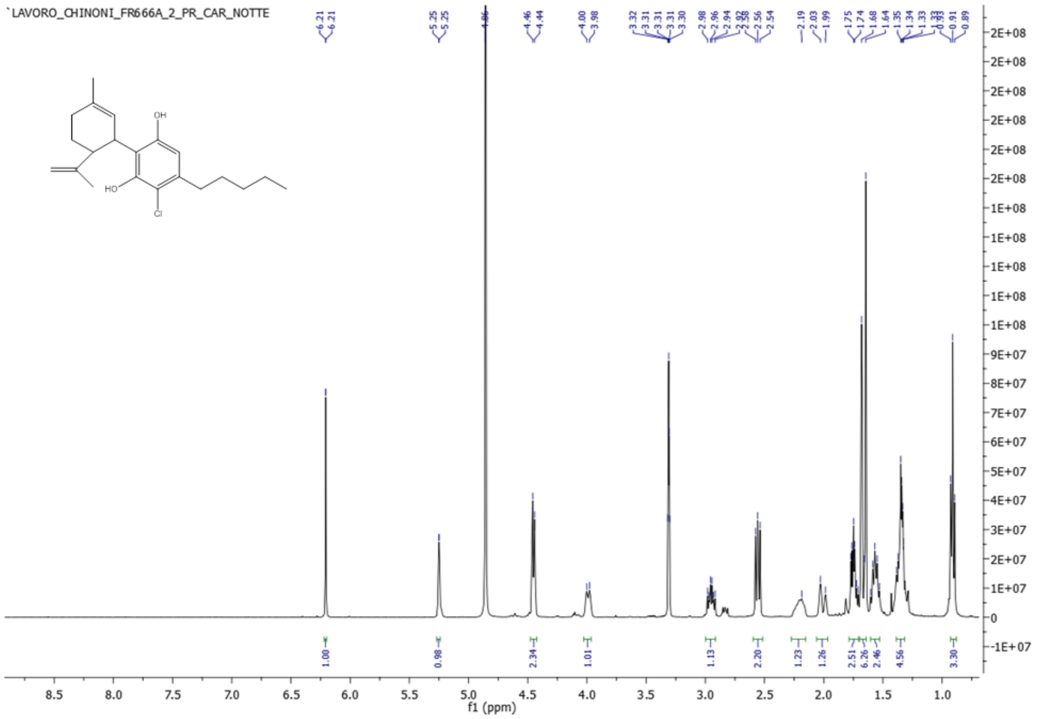

Figure S6. ^{1}H NMR spectrum (400 MHz) of compound **12** in CD_3OD

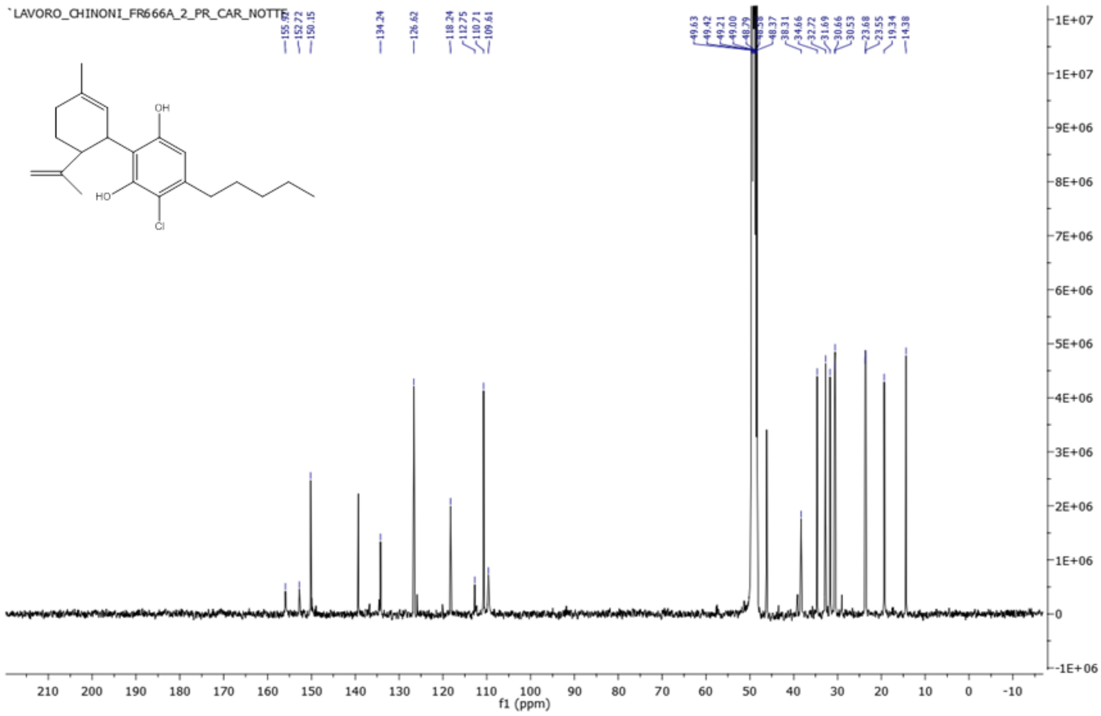

Figure S7. ^{13}C NMR spectrum of compound **12** in CD_3OD

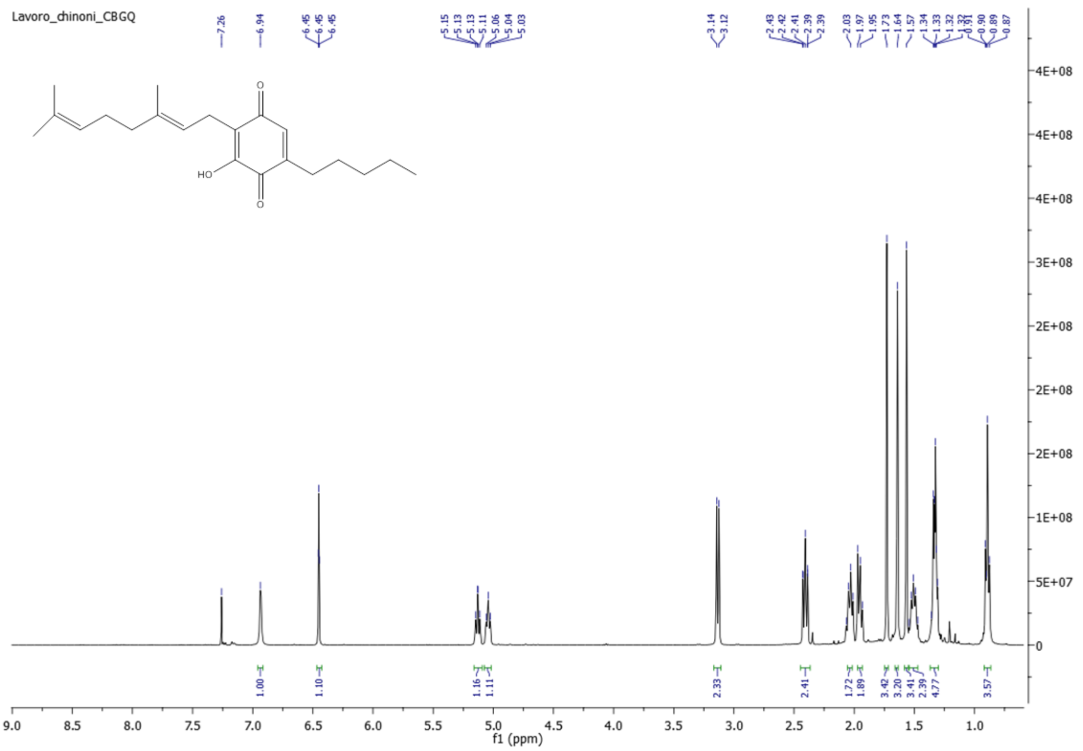

Figure S8. ^{1}H NMR spectrum (500 MHz) of compound **20** in $CDCl_3$

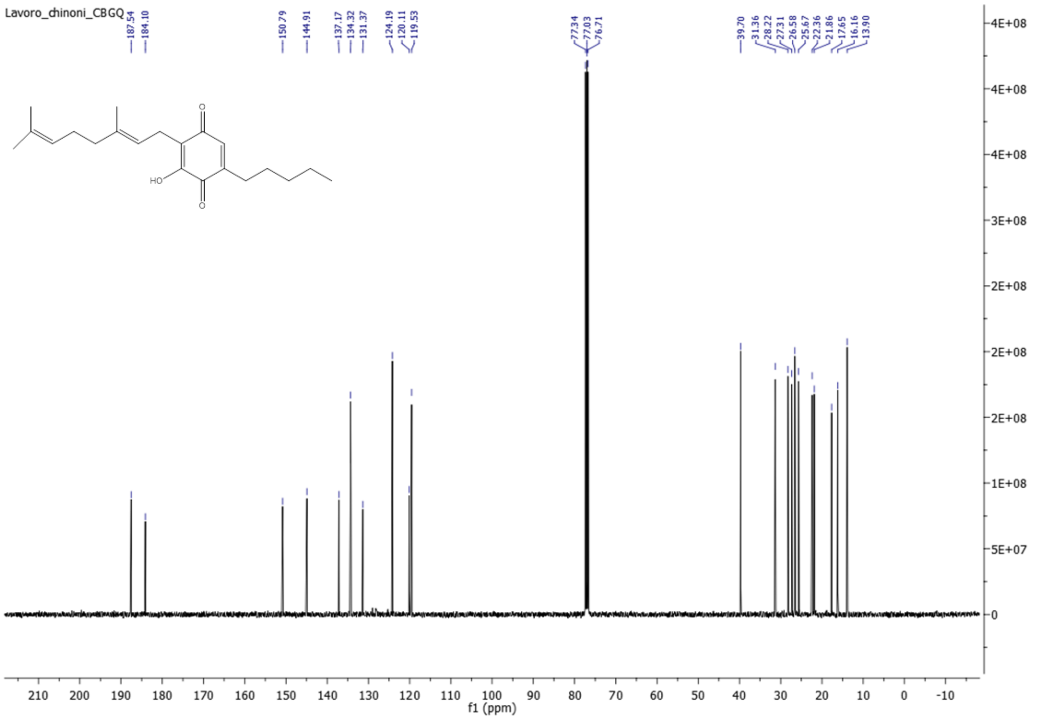

Figure S9. ^{13}C NMR spectrum of compound **20** in $CDCl_3$

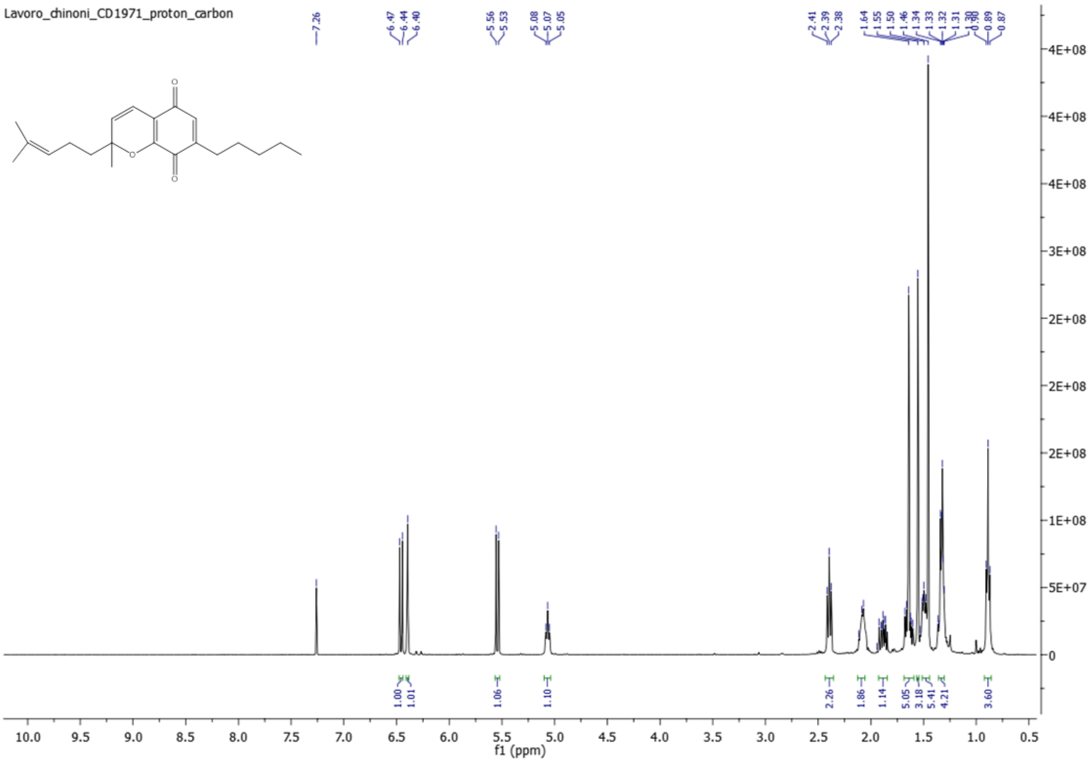

Figure S10. ^{1}H NMR spectrum (400 MHz) of compound **21** in $CDCl_3$

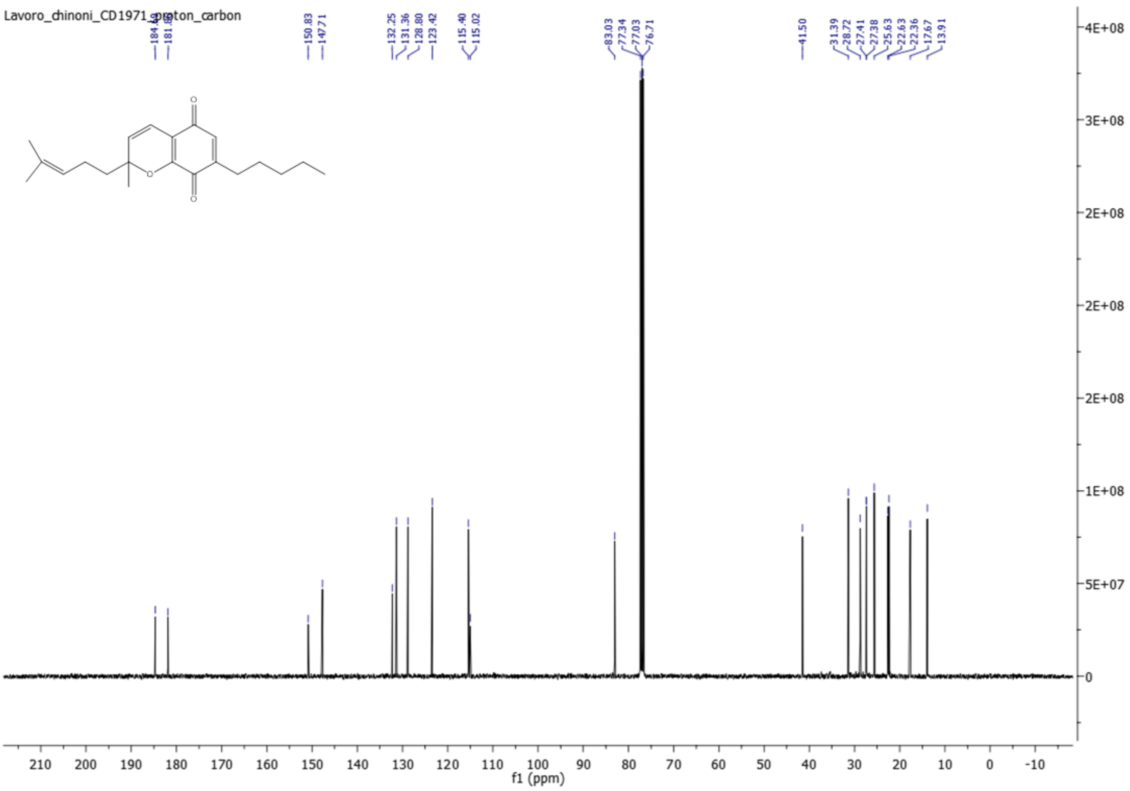

Figure S11. ^{13}C NMR spectrum of compound **21** in $CDCl_3$

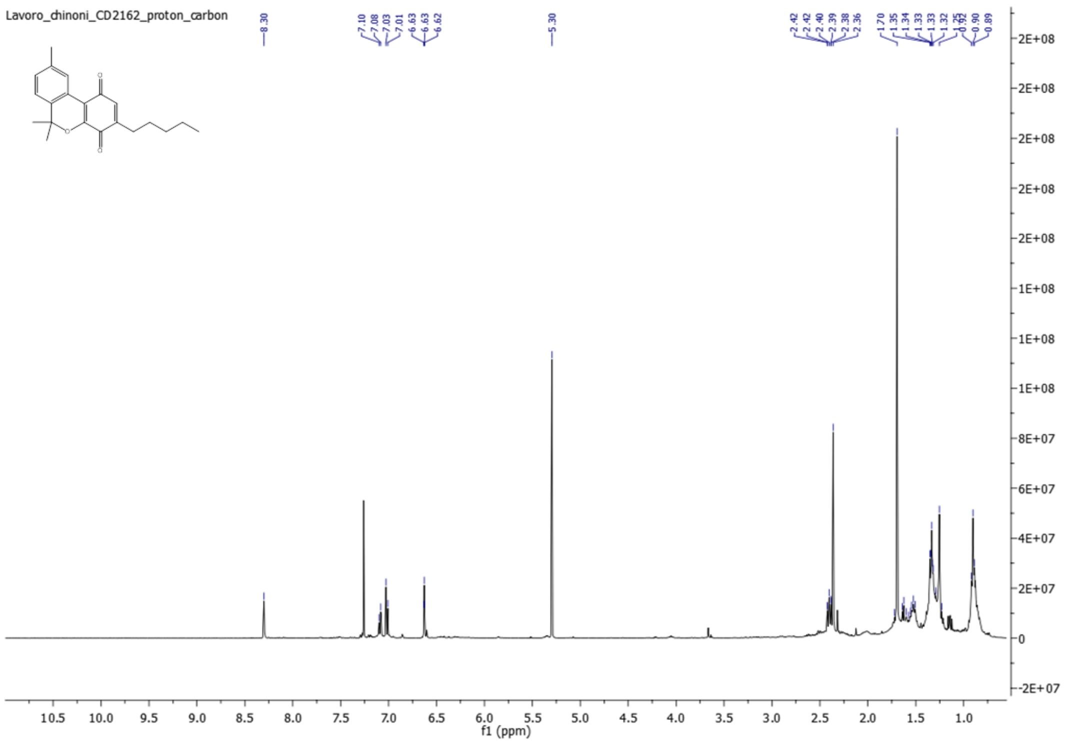

Figure S12. ^{1}H NMR spectrum (400 MHz) of compound **22** in $CDCl_3$

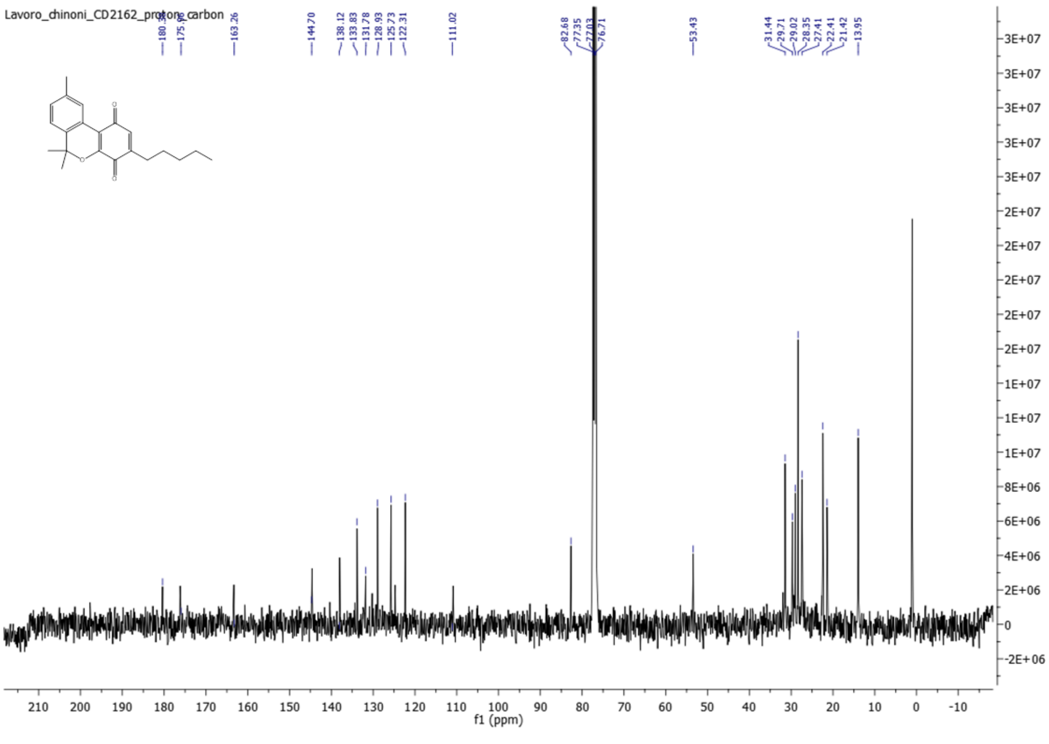

Figure S13. ^{13}C NMR spectrum of compound **22**

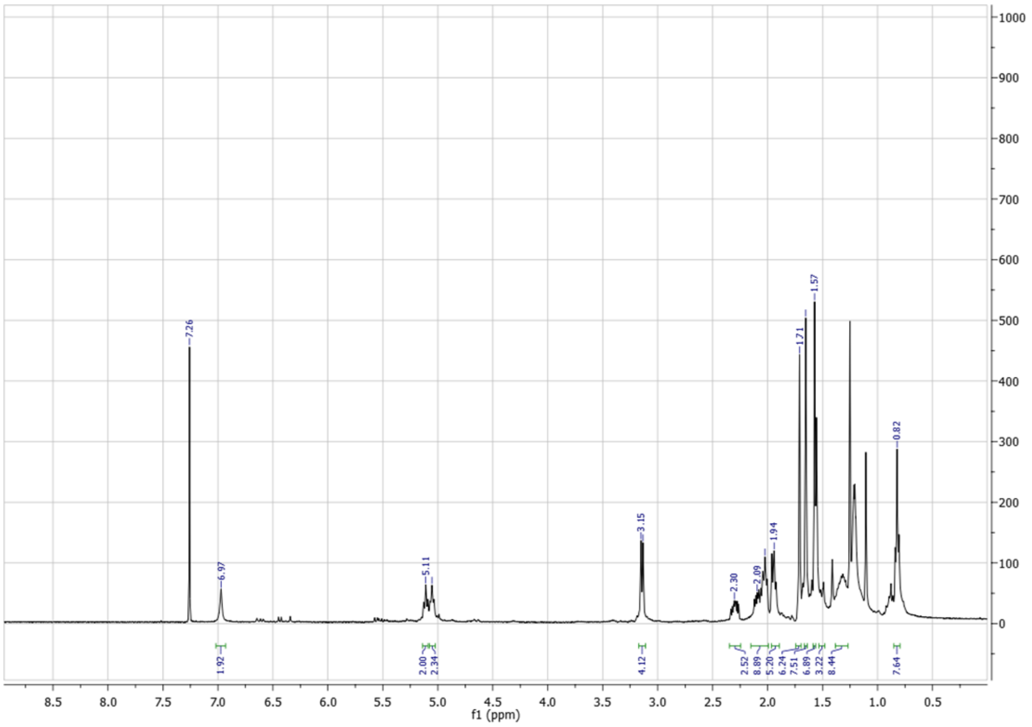

Figure S14. ^{1}H NMR spectrum (400 MHz) of compound **23** in $CDCl_3$

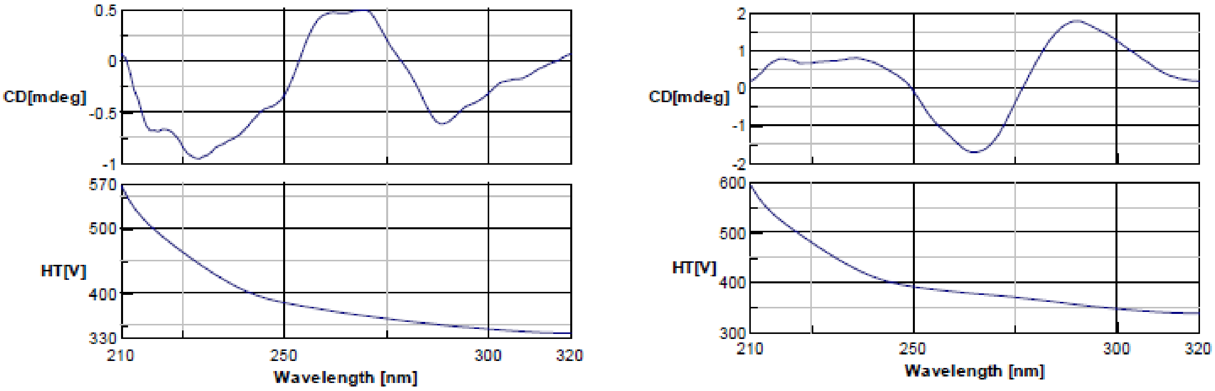

Figure S15. CD spectra of the two peaks obtained by chiral chromatography of compound **23**
